# Fusion of Motif- and Spectrum-Related Features for Improved EEG-Based Emotion Recognition

**DOI:** 10.1155/2019/3076324

**Published:** 2019-01-17

**Authors:** Abhishek Tiwari, Tiago H. Falk

**Affiliations:** Institut National de la Research Scientifique, Université du Québec, Montréal, Québec, Canada

## Abstract

Emotion recognition is a burgeoning field allowing for more natural human-machine interactions and interfaces. Electroencephalography (EEG) has shown to be a useful modality with which user emotional states can be measured and monitored, particularly primitives such as valence and arousal. In this paper, we propose the use of ordinal pattern analysis, also called motifs, for improved EEG-based emotion recognition. Motifs capture recurring structures in time series and are inherently robust to noise, thus are well suited for the task at hand. Several connectivity, asymmetry, and graph-theoretic features are proposed and extracted from the motifs to be used for affective state recognition. Experiments with a widely used public database are conducted, and results show the proposed features outperforming benchmark spectrum-based features, as well as other more recent nonmotif-based graph-theoretic features and amplitude modulation-based connectivity/asymmetry measures. Feature and score-level fusion suggest complementarity between the proposed and benchmark spectrum-based measures. When combined, the fused models can provide up to 9% improvement relative to benchmark features alone and up to 16% to nonmotif-based graph-theoretic features.

## 1. Introduction

Human-machine interaction can become more natural once machines become aware of their surroundings and their users [1, 2]. These so-called context-aware or affective interfaces can open up new dimensions of device functionality, thus more accurately addressing human needs while keeping the interfaces as natural as possible [3]. For example, affective computing can enable applications in which the machine can learn user preferences based on their reactions to different settings or even become a more effective tutor by assessing the student's emotional/stress states [3]. Automated recommender and tagging systems, in turn, can make use of affect information to better understand user preferences, thus improving system usability [4]. Measuring affective state and engagement levels can also be used by a machine to infer the user's perceived quality of experience [5–9], thus providing the machine with an objective criterion for online optimization.

Human emotions are usually conceived as physiological and physical responses and are part of natural human-human communications. Emotions are able to influence our intelligence, shape our thoughts, and govern our interpersonal relationships [10–13]. Emotion is usually expressed in a multimodal way, either verbally through emotional vocabulary or by expressing nonverbal cues such as intonation of voice, facial expressions, and gestures. As such, audio-visual cues have been widely used for affective state monitoring [14]. Alternately, emotions have also been known to effect neurophysiological signals; thus, biosignal monitoring has been extensively explored. Representative physiological signal modalities have included galvanic skin response (GSR), skin temperature, and breathing and cardiac activity (via electrocardiography (ECG) and photoplethysmography (PPG)) [15–18].

More recently, brain-computer interfaces (BCIs) have emerged as another tool to accurately monitor implicit user information, such as mood, stress level, and/or emotional states [9, 19, 20]. Within BCI-based affective computing methods, electroencephalography (EEG) has remained the most popular modality due to its noninvasiveness, high temporal resolution, and portability [21], though alternative modalities, such as near-infrared spectroscopy (NIRS), are slowly emerging [5, 22, 23]. Typically, with EEG-based systems, spectral power features have been widely used (e.g., [18, 24–26]), including frontal interhemispheric asymmetry features [27–32]. EEG signals, however, are very sensitive to artefacts, such as eye blinks and muscle movement [33]. To overcome such issues, artefact removal algorithms can be used. Alternately, new noise-robust features can be developed and/or multimodal fusion strategies can be explored [34].

In this paper, focus is placed on the latter and motif-based features are proposed and tested alone or alongside alternate complementary features. Motif-based analysis has shown to be useful in the past to recognize sleep states [35], as well as the effects of anesthesia [36], to detect seizures [37, 38], and to measure alertness [39]. Motif-based methods are inherently robust to noise as they deal with the shape of the time series and are unaffected by the magnitude [38, 40, 41]. To the best of our knowledge, they have yet to be explored for affective state monitoring; thus, this paper fills this gap. In particular, we compare the proposed features with spectral power and spectral asymmetry benchmark features. Notwithstanding, one main limitation of motif features concerns the loss of both amplitude and rate-of-change information when time series are converted into motif series [40, 42]. As such, we also explore three different fusion strategies to combine information from the proposed motif features and classical benchmark features. Experimental tests on a publicly available database [18] are performed, which show the advantages of the proposed features over benchmark ones, as well as the benefits of fusion for affective state monitoring.

The remainder of this paper is organized as follows.  Section 2 describes the materials and methods used, including the database considered, proposed, and benchmark features, fusion methods used, and performance metrics used.  Section 3 then presents and discusses the results obtained, and conclusions are drawn in Section 4.

## 2. Materials and Methods

Here, we describe the database used, benchmark features, proposed motif features, as well as the feature selection schemes employed, classifiers, and fusion schemes explored.

### 2.1. DEAP Database

This study relies on the publicly available, widely used DEAP (Dataset for Emotion Analysis using EEG and physiological signals) database. As detailed in [18], thirty-two healthy participants (50% females, average age = 26.9 years) were recruited and consented to participate in the study. Thirty-two channel EEG data were recorded using a Biosemi ActiveTwo system (Amsterdam, Netherlands) at a sampling rate of 512 Hz. Electrodes were placed on the scalp according to the International 10–20 system.

**Figure 1:**
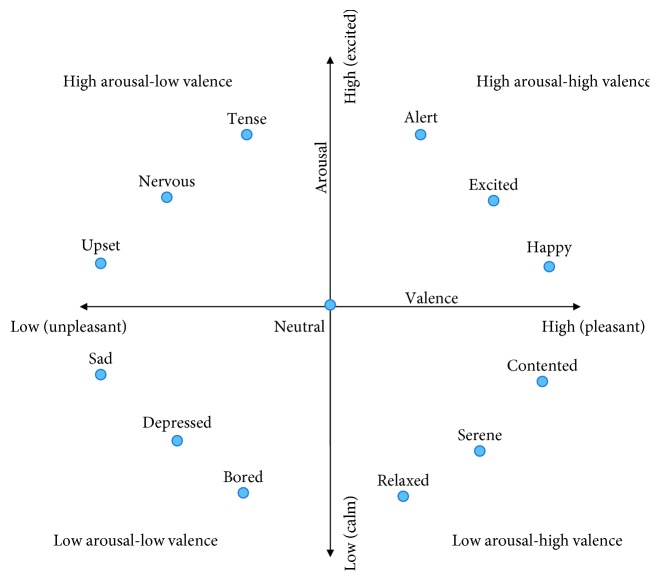
Valence-arousal plot with representative emotions.

Participants were presented with 40 one-minute long music videos with varying emotional content. These video clips were selected based on a previous analysis of several hundred videos as they were shown to elicit the strongest reactions across the four quadrants in the valence-arousal space (i.e., low valence, low arousal; low valence, high arousal; high valence, low arousal; and high valence, high arousal). The valence-arousal space is a two dimensional scale used to characterize emotions [43]. Valence refers to the (un)pleasantness of an event, whereas arousal refers to the intensity of the event, ranging from very calming to highly exciting. Using this space, various emotions can be mapped, as shown in Figure 1. Prior to each video, there was a baseline period of five seconds where the participants were asked to fixate at a cross in the middle of the screen. Following the presentation of each video, participants were asked to rate the music videos on discrete 9-point scales for valence and arousal using the self-assessment manikins (SAM) [44]. While other dimensional ratings, such as dominance and liking were also collected, these have not been explored herein.

The EEG data are available for public download in raw format or in preprocessed format, which includes common referencing, down-sampling to 128 Hz, bandpass filtering between 4 and 45 Hz, and eye blink artefact removal via independent component analysis. Moreover, only the last three seconds of the five-second baseline are available. Since this is a standard pipeline for EEG processing, the analysis reported herein is done on the preprocessed data. Data per subject were epoched into forty 60 s long trials with a 3 s long prestimulus baseline. The prestimulus baseline was then subtracted from the preprocessed data. The interested reader can refer to [18] for more details on the DEAP database and its data collection process.

### 2.2. Benchmark Features

As mentioned previously, spectral power features in different EEG bands have been widely used for affective state monitoring, including for the DEAP database [18, 45]. Moreover, an interhemispheric asymmetry in spectral power has also been been reported in the affective state literature [27, 28, 30–32], particularly in frontal brain regions [29, 31]. Typically, EEG signals are band decomposed into theta (4 < *θ* < 8  Hz), alpha (8 < *α* < 13  Hz), beta (13 < *β* < 30  Hz), and gamma (30 < *γ* < 45  Hz) bands. Here, 48 asymmetry index (AI) features (12 interhemisphereic electrode pairs × 4 bands) were computed for the following electrodes pairs: Fp2-Fp1, F3-F4, F7-F8, FC1-FC2, FC5-FC6, C3-C4, T7-T8, Cp1-Cp2, Cp5-Cp6, P3-P4, P7-P8, and O1-O2.

Moreover, EEG band ratios have also been explored in the past for tasks such as human mental state monitoring, fatigue, attention control, and negative emotional response monitoring [46–48], thus are also included here as benchmark features. The ratios computed include *γ*/*β*, *β*/*θ*, *α*/*θ*, (*α*+*β*)/*γ*, and  (*γ*+*β*)/*θ*. The ratios are computed individually over each electrode. Lastly, the Shannon entropy [49] has been used as a feature to measure the complexity of the EEG time series. Shannon entropy can be calculated as follows:

(1) $$\begin{array}{c} \text{SE}=-\underset{j}{\sum }P_{j}\cdot \text{log}\left(P_{j}\right), \end{array}$$

where *P*_*j*_ is the power in sub-band *j*.

### 2.3. Motif-Based Features

A motif is a pattern or structure characterized by the number of nodes or degree (represented by *n*) and the connection between them and the number of points used between these nodes (called lag, represented by *λ*). Each motif can be represented as an alphabet or a number. The robustness of motif features comes from the fact that they only consider the underlying shape of the time series and not the amplitude. Using this definition, any time series *X*(*i*) can be converted into a motif series *X*_*m*_(*i*) using these given rules (e.g., for degree, *n*=3):

(2) $$\begin{array}{c} X_{m}\left(i\right)=\left\{\begin{array}{cc} 1 & \text{if }X\left(i\right)<X\left(i+\lambda \right)\;X\left(i+\lambda \right)<X\left(i+2\lambda \right)\;X\left(i\right)<X\left(i+2\lambda \right),\\ 2 & \text{if }X\left(i\right)<X\left(i+\lambda \right)\;X\left(i+\lambda \right)>X\left(i+2\lambda \right)\;X\left(i\right)<X\left(i+2\lambda \right),\\ 3 & \text{if }X\left(i\right)>X\left(i+\lambda \right)\;X\left(i+\lambda \right)<X\left(i+2\lambda \right)\;X\left(i\right)<X\left(i+2\lambda \right),\\ 4 & \text{if }X\left(i\right)<X\left(i+\lambda \right)\;X\left(i+\lambda \right)>X\left(i+2\lambda \right)\;X\left(i\right)>X\left(i+2\lambda \right),\\ 5 & \text{if }X\left(i\right)>X\left(i+\lambda \right)\;X\left(i+\lambda \right)>X\left(i+2\lambda \right)\;X\left(i\right)>X\left(i+2\lambda \right),\\ 6 & \text{if }X\left(i\right)>X\left(i+\lambda \right)\;X\left(i+\lambda \right)<X\left(i+2\lambda \right)\;X\left(i\right)>X\left(i+2\lambda \right). \end{array}\right. \end{array}$$

**Figure 2:**
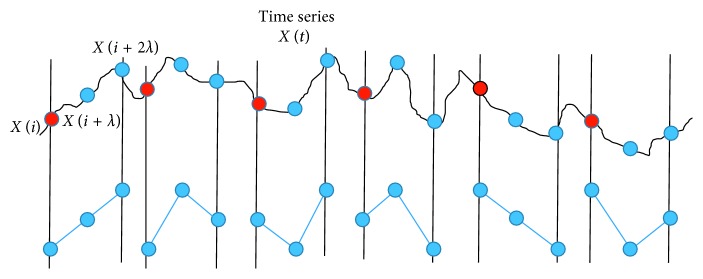
All motifs of degree *n*=3 appearing in a time series.

Figure 2 shows the different motifs possible for degree *n*=3 appearing in a particular time series. Once the motif series has been derived, different features can be extracted based on the statistics of recurring patterns within the motif series. The features proposed herein are detailed in the subsections below and only consider motifs of degree *n*=3 and lag value *λ*=1. These parameters have been suggested in the past for related tasks [39, 50].

#### 2.3.1. Permutation Entropy

Permutation entropy (PE) [41] is a commonly derived motif-based metric and is calculated as

(3) $$\begin{array}{c} \text{PE}=-\overset{n!}{\underset{j}{\sum }}p\left(j\right)\cdot \text{log}\left(p\left(j\right)\right), \end{array}$$

where *p*(*j*) is the relative frequency of the motif pattern represented by *j*.

#### 2.3.2. Ordinal Distance Dissimilarity

Ordinal distance-based dissimilarity [38] is a metric with close parallel to the benchmark asymmetry index and measures the dissimilarity between two motif series for different electrode pairs using

(4) $$\begin{array}{c} D_{m}\left(X,Y\right)=\sqrt{\frac{n!}{n!-1}}\sqrt{\overset{n!}{\underset{i}{\sum }}{\left(p_{x}\left(i\right)-p_{y}\left(i\right)\right)}^{2}}, \end{array}$$

where *p*_*x*_(*i*) and *p*_*y*_(*i*) are the relative frequencies of the motif pattern represented by *i* in electrodes *X* and *Y*, respectively, and *n* is the degree of the motif. In order to compare against the benchmark asymmetry index, ordinal dissimilarity is calculated for the same electrode pairs reported in Section 2.2.

#### 2.3.3. Motif Synchronization

Functional connectivity gives insight into the dynamic neural interaction of the different regions of the brain. Recently, motif synchronization has been proposed as a functional connectivity analysis tool [50] and measures the simultaneous appearance of motifs in two time series. For two motif series *X*_*m*_ and *Y*_*m*_, *c*(*X*_*m*_; *Y*_*m*_) is defined as the highest number of times in which the same motif can appear in *Y*_*m*_ shortly after it appeared in *X*_*m*_ for different delay times, i.e.,

(5) $$\begin{array}{c} c\left(X_{m};Y_{m}\right)=c_{XY}=\text{max}\left(\overset{l_{m}}{\underset{i=1}{\sum }}J_{i}^{\tau_{0}},\overset{l_{m}}{\underset{i=1}{\sum }}J_{i}^{\tau_{1}},\ldots ,\overset{l_{m}}{\underset{i=1}{\sum }}J_{i}^{\tau_{n}}\right), \end{array}$$

with

(6) $$\begin{array}{c} J_{i}^{\tau }\left(i\right)=\left\{\begin{array}{cc} 1, & \text{if }X_{M_{i}}=Y_{M_{i+\tau }}\;,\\ 0, & \text{else}. \end{array}\right.. \end{array}$$

The time delay *τ* ranges from *τ*_0_=0 to *τ*_*n*_, where *τ*_*n*_ is the maximum value to be considered, and *l*_*m*_ is the size of the time varying window within the time series. Similarly, the opposite measure *c*_*YX*_ can be obtained by changing only the order of the time series to *Y*_*M*_*i*__=*X*_*M*_*i*+*τ*__. Finally, the degree of synchronization *Q*_*XY*_ and the synchronization direction *q*_*XY*_ are given by

(7) $$\begin{array}{c} Q_{xy}=\frac{\text{max}\left(c_{XY},c_{YX}\right)}{l_{m}},\\ q_{XY}=\left\{\begin{array}{cc} 0, & \text{if}\;\;c_{XY}=c_{YX},\\ \mathrm{sign}\left(c_{XY}-c_{YX}\right), & \text{else}. \end{array}\right. \end{array}$$

The degree of synchronization, *Q*_*XY*_, is scaled between 0 and 1, with 0 representing no interaction and 1 suggesting very high interactions. Feature *q*_*XY*_, in turn, gives the direction of information flow, with 0 indicating no preferred direction, 1 indicating direction from *X* to *Y*, and −1 indicating direction from *Y* to *X*. For our calculation, *τ*_*n*_ has been chosen as 5 and the window size *l*_*m*_ is chosen as 256.

#### 2.3.4. Graph Features

The different functional connections obtained by motif synchronization analysis can be further extended by means of graph-theoric analysis, where each electrode on the scalp represents a node on the brain network. Weighted graphs have weights that represent the level of interaction between the two nodes. Edges with smaller weights are believed to represent noisy/spurious connections [51], thus a thresholding is done to obtain an unweighted graph. Previously, graph-theoretic features have been explored for affect recognition based on EEG spectral coherence measures [52]. Graph-theoretic analysis based on motifs, however, has yet to be explored, thus both weighted and unweighted graphs (thresholded to the average value of the graph weighted) are tested herein. An advantage of motif synchronization over more popular connectivity approaches is the ability it provides to measure direction of information flow for the different nodes in the brain network. From the weighted and unweighted graphs, several features are extracted, namely,

(i) Degree of connectivity (*k*): the degree of connectivity is defined as *k*_*i*_ where *i* is a given node. For the unweighted network, it is calculated as
  (8) $$\begin{array}{c} k_{i}=\underset{j\epsilon N_{e}}{\sum }a_{ij}, \end{array}$$
  where *N*_*e*_ represents the nodes in the network and *a*_*ij*_, *i* ≠ *j* represents the value of the unweighted adjacency matrix. For the weighted network, the formula is
  (9) $$\begin{array}{c} k_{i}^{w}=\underset{j\epsilon N_{e}}{\sum }w_{ij}, \end{array}$$
  where instead of *a*_*ij*_, the weights *w*_*ij*_ assigned to each edge are used. The average degree of connectivity for the whole network is used as a feature in our analysis.
(ii) Clustering coefficient (*C*): the mean clustering coefficient for an unweighted network is given by
  (10) $$\begin{array}{c} C=\frac{1}{N_{e}}\overset{N_{e}}{\underset{i}{\sum }}\frac{e_{i}}{k_{i}\left(k_{i}-1\right)}, \end{array}$$
  where *e*_*i*_ is the number of existing edges between the neighbors of *i* and *k*_*i*_ is the degree of connectivity for the unweighted network. For a weighted network, the clustering coefficient value is given by
  (11) $$\begin{array}{c} C^{w}=\frac{1}{N_{e}}\overset{N_{e}}{\underset{i}{\sum }}\frac{t_{i}^{w}}{k_{i}^{w}\left(k_{i}^{w}-1\right)}, \end{array}$$
  where *t*_*i*_ is calculated as
  (12) $$\begin{array}{c} t_{i}=\frac{1}{2}\underset{j,h\epsilon N_{e}}{\sum }{\left(w_{ij}w_{jh}w_{hi}\right)}^{1/3}, \end{array}$$
  and represents the geometric mean of the triangles constructed from the edges around a particular node *i*, and *k*_*i*_^*w*^ represents the weighted degree of connectivity.
(iii) Transitivity (Tr): transitivity is defined as the ratio of “triangles to triplets” in a network and is defined as
  (13) $$\begin{array}{c} \text{Tr}=\frac{3\lambda }{k\left(k-1\right)}, \end{array}$$
  where *λ* represents the number of triangles in network, while *k* is the average degree of connectivity (weighted or unweighted) of a network. Transitivity is a global measure of the clustering coefficient and is equal to it when the degree of connectivity of all nodes is equal to one another.
(iv) Characteristic path length (*L*): for an unweighted network, *L* is given by
  (14) $$\begin{array}{c} L=\frac{1}{N_{e}\left(N_{e}-1\right)}\overset{N_{e}}{\underset{j=1i\neq j}{\sum }}d_{ij}, \end{array}$$
  with *d*_*ij*_ being the minimum amount of edges required to connect nodes *i* and *j* and is replaced by the shortest weighted path length *d*_*ij*_^*w*^ for the weighted characteristic path length *L* ^*w*^.
(v) Global efficiency (*G*): this is calculated using the inverse of the shortest weighted or unweighted path for the network, i.e.,
  (15) $$\begin{array}{c} G=\frac{1}{N_{e}\left(N_{e}-1\right)}\overset{N_{e}}{\underset{j=1i\neq j}{\sum }}d_{ij}^{-1}, \end{array}$$
  where *d*_*i*_ *j* is replaced by the shortest weighted path length *d*_*ij*_^*w*^ for weighted global efficiency measure.
(vi) Small-world features: the work in [53] has shown that human brain networks exhibit small-world characteristics. A small-world network is characterized by a high clustering coefficient and a small average path length from one node to another [54]. Here, three small-world features are computed, namely, (i) the small-world characteristics length:
  (16) $$\begin{array}{c} L_{s}=\frac{L}{L_{\text{rand}}}, \end{array}$$
  (ii) the small-world clustering coefficient:
  (17) $$\begin{array}{c} C_{s}=\frac{C}{C_{\text{rand}}}, \end{array}$$
  and (iii) the small-worldness of a network [55]:
  (18) $$\begin{array}{c} S=\frac{C_{s}}{L_{s}}, \end{array}$$
  where *C*_rand_ and *L*_rand_ are the corresponding clustering coefficient and characteristic path length values for a random network, respectively.
(vii) Direction of flow (DoF): As motif synchronization also provides the direction of information flow in the brain network graph, a simple feature is explored here to represent the overall response of the brain network as either receiving or transmitting information, on average. DoF is defined as
  (19) $$\begin{array}{c} \text{DoF}=\underset{ij}{\sum }q_{ij}, \end{array}$$
  where *q*_*ij*_ is defined as the direction of information flow with 1 representing information flowing from *i* to *j*, −1 representing information flow in the opposite direction, and 0 being no preferred information flow direction.

Table 1 provides a summary of the number of features extracted for each feature group and subgroup.

### 2.4. Feature Selection

Previous work has shown that motif features convey complementary information to other amplitude- and rate-of-change-based features [40, 42]. As such, we explore the effects of combining the proposed motif-based features with the benchmark ones. Given the small dataset size, however, it is important to avoid issues with curse of dimensionality and overfitting; thus, feature selection is required. Here, three feature selection strategies have been explored:

1. ANOVA-based feature ranking and selection: this selection method is based on calculating the significance of the input features with respect to the output values and returning the ranked features according to their obtained *p* values.
2. Minimum redundancy maximum relevance (mRMR) feature selection: the mRMR is a mutual information-based algorithm that optimizes two criteria simultaneously: the maximum-relevance criterion (i.e., maximizes the average mutual information between each feature and the target vector) and the minimum-redundancy criterion (i.e., minimizes the average mutual information between two chosen features). The algorithm finds near-optimal features using forward selection with the chosen features maximizing the combined max-min criteria. Previous work showed mRMR paired with a support vector machine (SVM) classifier [56] achieved the best performance in EEG-based emotion recognition tasks [57].
3. Recursive feature elimination (RFE): given an external estimator that assigns weights to features, the least important features are pruned from the current set of features. The procedure is recursively repeated on the pruned set until the desired number of features to select is reached. This technique considers the interaction of features with the learning algorithm to give the optimal subset of features. Since recursive training and feature elimination is required, this method takes a significant amount of runtime.

For the experiments herein, 90% of the data is set aside for feature selection and classifier training and the remaining 10% is left aside for testing. The split was performed with a random seed of 0 using the scikit-learn function in Python. The best feature selection algorithm and its corresponding optimal number of features are then selected by grid search. Classifier training and different fusion schemes are described next.

### 2.5. Classification

SVMs have been widely used for affective state recognition [57] and are explored herein as well. Given their widespread use, a detailed description is beyond the scope of this paper and the interested reader is referred to [58] and references therein for more details. Here, SVM classifiers are trained on two different binary classification problems, namely, discriminating between low and high valence states and low and high arousal states. For our study, a radial basis function (RBF) kernel was used and implemented with the scikit-learn library in Python [59]. As we are interested in exploring the benefits of the proposed motif features and comparing them against benchmark features, we do not perform classifer hyperparameter optimization and use default parameters instead, namely, *λ*_SVM_=1 and *γ*_RBF_=0.01.

**Figure 3:**
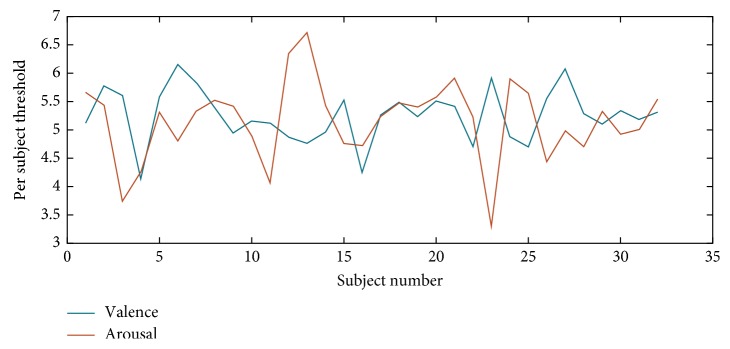
Threshold variation for each subject for valence (blue) and arousal (orange) dimensions.

Moreover, as the DEAP database relies on 9-point scale ratings, it has typically been the case where the midpoint is considered as a threshold, where ratings greater than the threshold are considered “high,” and those below are considered “low”. As was recently emphasized in [4], however, subjects have their own internal biases, thus leading to varying scales for grading and, consequently, different thresholds per participant. For example, as reported in [4], by using a midpoint threshold value of 5, a 60/40 ratio of high/low levels was obtained across all participants. In turn, if an individualized threshold was used corresponding to the value in which an almost-balanced high/low ratio was achieved per participant, improved results were achieved [60].  Figure 3, for example, depicts the threshold found for each participant for arousal and valence in this latter scenario. As can be seen, on average, a threshold of 5 was most often selected, though in a few cases, much higher or much lower values were found, thus exemplifying the need for the individualized approach used herein.

### 2.6. Fusion Strategies

Here, we explore three different types of fusion strategies to combine motif-based and benchmark spectrum-based features, which are described below.

#### 2.6.1. Feature Fusion

As the name suggests, this corresponds to the direct combination of motif and benchmark features prior to feature selection.

#### 2.6.2. Score-Level Fusion

The weighted decision fusion method proposed in [61] has been used. According to this technique, the fusion classification probability *p*_0_^*x*^ for *x* *ϵ* [0,1] for each class *x* *ϵ* 1,2 can be denoted by

(20) $$\begin{array}{c} p_{0}^{x}=\overset{N}{\underset{i=1}{\sum }}\alpha_{i}p_{i}^{x}t_{i}, \end{array}$$

where *i* is the index of a particular feature group, *N* is the total number of groups used, and *α*_*i*_ are the weights corresponding to each group (∑_*i*_^*N*^ *α*_*i*_=1). The parameter *t*_*i*_ is the training set performance of a particular feature group such that the fusion probabilities for all classes sum up to unity and is given by

(21) $$\begin{array}{c} t_{i}=\frac{F_{i}}{{\sum^{}}_{i}^{N}\alpha_{i}F_{i}}, \end{array}$$

where, *F*1 is the *F*1-score obtained on the training set using a particular feature group. The weight space was searched for best performance as this is indicative of the contribution to the outcome made by each of the feature groups.

#### 2.6.3. Output Associative Fusion

**Figure 4:**
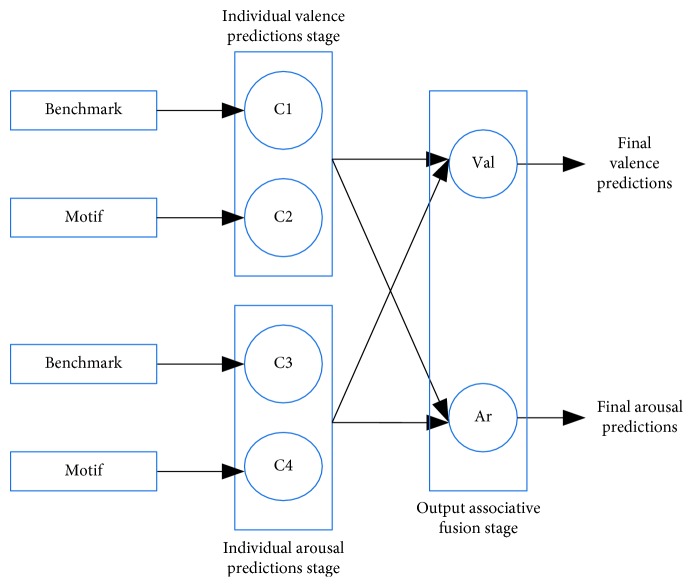
Block diagram of OAF strategy for the two feature groups.

Psychological evidence has suggested a strong intercorrelation between the valence and arousal dimensions [62–65]. As such, the output associative fusion (OAF) method has been used to model the correlations for continuous prediction of valence and arousal scales [66]. The OAF framework has been explored here and is depicted by the block diagram in Figure 4. As can be seen, first individual classifiers make the valence and arousal predictions for each individual feature group. This is then followed by a final prediction step which considers both the valence and arousal dimensions in order to better predict each of the two outputs.

### 2.7. Figure of Merit

Balanced accuracy (BACC) has been used as the performance metric as it takes into account class unbalances. Balanced accuracy corresponds to the arithmetic mean of the classifier sensitivity and specificity, i.e.,

(22) $$\begin{array}{c} \text{BACC}=\frac{\text{sens}+\text{spec}}{2}, \end{array}$$

where

(23) $$\begin{array}{c} \text{sens}=\frac{\text{TP}}{P},\\ \text{spec}=\frac{\text{TN}}{N}, \end{array}$$

with *P*=TP+FN and *N*=FP+TN, and TP and FP correspond to true and false positives, respectively, while TN and FN correspond to true and false negatives.

To test the significance of the attained performances against chance, an independent one-sample *t*-test against a random voting classifier was used (*p* ≤ 0.05), as suggested in [18]. In order to have a more generalized performance of the classifier, once the feature selection step is performed, classifier training and testing are performed 100 times with different train/test partitioning. This setup provides a more generalized performance of the features and their invariance to the training set used. The BACC values reported in the tables correspond to the mean ± the standard deviation of all BACC values attained on the test set over all of the 100 iterations.

## 3. Results and Discussion

In this section, we show and discuss the obtained results in terms of impact of feature selection, feature group, and fusion strategy on overall performance.

### 3.1. Feature Selection

As mentioned previously, three different feature selection schemes were explored and tested herein. Feature selection was implemented in the benchmark features alone, proposed motif feature alone, and in the combined benchmark-motif set. The optimal BACC values obtained are shown in Tables 2 [Table tab3]–4, respectively, along with the final number of features (nofs) used in the models.

As can be seen, for ANOVA-based feature selection, fewer than 10 features were used in the models for both valence and arousal dimensions with the benchmark features, thus representing roughly one-sixth of the total amount of available features. For the motif group, in turn, roughly 40 were shown to be useful, thus amounting to roughly one-third of the available feature pool. When combining both feature sets, the optimal model also relied on roughly 40 features, thus one quarter of the available feature pool.

The mRMR algorithm, in turn, generally resulted in fewer top features but with similar overall BACC, thus corroborating the results in [56, 57]. For the benchmark feature set, for example, BACC ≈ 0.54 was achieved with just three features for valence, thus in line with the ≈0.55 achieved with ANOVA-selected features. For arousal and motif features, similar BACC was achieved, but relying on roughly half the number of features relative to ANOVA-based selection. With the combined feature set, in fact, improved BACC was achieved for the arousal dimension but with fewer than half the number of features chosen by ANOVA.

Lastly, RFE selection typically resulted in the highest accuracy with the best BACC vs. nof tradeoff. This is expected as RFE considers the interaction of features among themselves and the final outcome. Overall, the best accuracy was achieved with the combined set, followed closely by the models trained on the proposed motif features. These findings corroborate the complementarity of the two different feature types and show the importance of motif features for affective state recognition.

A one-way ANOVA was computed between the different pairs of feature selection algorithms (ANOVA vs. mRMR, ANOVA vs. RFE, and RFE vs. mRMR) for the benchmark, motif, and combined feature sets to assess the algorithm performance. For the benchmark feature set, in the arousal dimension, the three algorithms perform similarly with no statistical differences observable. However, for the valence dimension, the RFE performs significantly better than the mRMR algorithm (*p*_val_ < 0.05), while there are no significant differences observed between RFE and ANOVA performances, the RFE obtains a similar performance with fewer features. For the motif feature set, in the arousal dimension, we observe the RFE performs significantly better than both ANOVA (*p*_val_ < 0.01) and mRMR (*p*_val_ ≈ 0.01). In the valence dimension, we observe a significant difference in algorithm performance between RFE and mRMR; however, the performance of ANOVA is not significant compared to both the algorithms. However, we again observe that RFE gives similar performance to ANOVA with half the number of features, thus being more efficient. Finally, for the combined feature set, in the arousal dimension, both mRMR and RFE perform significantly better than ANOVA (*p*_val_ < 0.01) while there are no differences between mRMR and RFE performances with mRMR reaching equivalent performance with fewer features than RFE. In the valence dimension, we observe ANOVA (*p*_val_ ≈ 0.05) and RFE (*p*_val_ < 0.01) perform significantly better than mRMR, while there is no performance difference between ANOVA and RFE in this case. It is interesting to note that the number of features for both ANOVA and RFE is almost the same. In general, we find the RFE gives significant or equal performance compared to ANOVA and mRMR with fewer number of features. For feature fusion, the algorithm giving the highest average performance has been considered the algorithm of choice.

Tables 5 and 6, in turn, report the top 20 features used in the models that achieved the best BACC for valence and arousal, respectively. As can be seen for valence (Table 5), the *γ*/*β* and *β*/*θ* power ratios showed to be important, along with alpha-band spectral power. This corroborates previous work which has linked *γ*/*β* and *β*/*θ* to audio comprehension [67, 68] and, consequently, to perceived valence in low-quality text-to-speech systems [5]. For the motif-based features, in turn, small-worldness (*γ* and *β* band) and weighted graph features (*θ* band) showed to be important, alongside PE for *γ* and *β* bands. Previous studies have indicated to a time-locked theta-band synchronization occurring during affective picture processing [69] related to the valence dimension. This synchronization seems to be captured by motif-based graph-theoretic and ordinal similarity features, as eight of the top 20 features come from the *θ* band.

Lastly, for the combined feature set, it can be seen that a mix of benchmark and motif features are selected, thus exemplifying the complementarity of the two feature sets. Over the entire nof=38 features used in the model, 11 are benchmark features and 27 are motif-based features. In particular, 17 of the top motif features showed importance across the motif and combined sets, as well as all of the top benchmark features across benchmark and combined sets. Additionally, for the combined set, 6 asymmetry features are also in the top selected features; of these, 3 are from the same electrode pairs as the top ordinal dissimilarity measures, thus showing a complementary nature of the two feature sets. The power ratios *α*/*θ*  and(*γ*+*β*)/*θ* also appear in the combined feature sets. From the motif feature sets, apart from the overlapping features, additional *D*_*m*_ and clustering coefficient features appear in the combined feature set along with two DoF features from the *θ* and *γ* bands.

For arousal (Table 6) and benchmark feature set, almost all power ratios showed to be important alongside several asymmetry index features, particularly those in the frontal and parietal regions. Such findings corroborate previous literature showing the relationship between (i) arousal and frontal asymmetry [29] in alpha band (e.g., [70]) and other bands (e.g., [71]), (ii) an inherent asymmetry in the right parietal-temporal regions, responsible for modulating autonomic and behavioural arousal, and (iii) arousal and EEG band power ratios [72].

For motif-based features, in turn, roughly half the top features corresponded to ordinal distance dissimilarity measures, thus corroborating the literature on EEG asymmetry and arousal [71, 73]. Moreover, the majority of the top features are from the beta and alpha bands (13 of the top 16), which have been linked to attention-based arousal changes [74] and to changes in visual selective attention [75, 76], which is very closely linked to arousal [77].

Interestingly, for the combined sets, none of the top features were from the benchmark feature set, thus suggesting that the proposed motif features conveyed improved arousal information relative to benchmark features. The majority of the features corresponded to ordinal distance dissimilarity across all EEG bands. Moreover, the best achieving model for motif only and combined feature sets were attained using different feature selection algorithms (RFE and mRMR, respectively). Notwithstanding, two features coincided as being important, namely, PE(*θ*), *D*_*m*_(*T*7, *T*8)(*α*), and a third showed similar behaviour (*C*(*α*) and *C* ^*w*^(*α*)), thus suggesting their importance for arousal prediction. In the combined set, *θ* showed up in seven of the nof=17, thus also corroborating previous findings [71, 73]. Lastly, most of ordinal dissimilarity features come from frontal, parietal, or temporal regions, thus in line with previous research connecting parietal-temporal regions with autonomic and behavioural arousal, as well as frontal regions with arousal [78].

### 3.2. Individual Feature Groups

So far, we have explored the performance achieved with benchmark, motif, and combined feature sets. It is interesting, however, to gauge how each individual feature subgroup contributes toward affective state recognition.  Table 7 reports the balanced accuracy for each individual feature subgroup for the best achieving model found after RFE feature selection.

As can be seen, for valence, the weighted and unweighted graph features achieve similar performances though the model based on the former feature subgroup relies on nof=2, as opposed to nof=8. In fact, all motif-based features achieved similar performance, with small-worldness features being the only ones not significantly better than the benchmark (i.e., *p* < 0.01 and indicated by an asterisk in the table). For arousal, in turn, it is observed that graph and small-world feature subgroups do not significantly improve over the benchmark, whereas other motif features, such as permutation entropy and ordinal distance dissimilarity, do. Overall, models relying on these two feature subgroups showed to provide the most discriminatory information for valence and arousal models.

Additionally, among the EEG features, we observe that SE, *θ*, and *γ* spectral power never appear as top selected features. This could be due to the fact that power and entropy measures are averaged over all electrodes, thus removing any spatial information relevant for the features. Notwithstanding, averaging ensures that the proposed features are invariant and robust to the electrode set considered, as seen with the global graph-theoretic features using motif synchronization. For valence, in turn, we observe that none of the AI features show up among the top in the EEG feature set alone scenario. When using only motif features, on the other hand, seven *D*_*m*_ features (out of nof = 20) are selected, thus suggesting that motif features may carry more relevant asymmetry signatures for the task at hand. With the combined feature set, it can be seen that proposed features from all groups appear in the top list for both valence and arousal.

### 3.3. Fusion Strategies

As mentioned previously, three fusion schemes were explored: feature, score, and output associative fusion. Tables 2 [Table tab3]–4 show the effects of feature fusion and the gains attained with the combined set relative to using only a feature group individually. For the valence dimension, for example, gains of 8.6% and 2.4% were achieved with feature fusion relative to using benchmark and motif feature alone, respectively. As shown in Table 5, the model based on the combined set relied on features from both feature groups, thus emphasizing their complementarity for valence prediction.

For arousal, feature fusion resulted in more modest gains relative to the benchmark (i.e., 6.1%) and to motif features (2.6%). Interestingly, the best model relied on mRMR selected features which did not include benchmark ones. The second best model, on the other hand, was achieved with RFE feature selection and the top 20 features included seven benchmark ones (i.e., (*α*+*β*)/*γ*, *β*/*θ*, AI(01, 02) (*β*), AI(Fc1, Fc2) (*β*), AI(C3, C4) (*γ*), AI(F3, F4) (*β*), and AI(Fc1, Fc2) (*γ*)), three of which overlap with the top features selected from the benchmark alone set. The remaining 13 features were from the motif group, nine of which showed to be top features selected in the motif alone set, namely, PE(*β*), PE(*θ*), *L* ^*w*^(*α*), *L* ^*w*^(*γ*), *C*_*s*_(*β*), *D*_*m*_(*P*7, *P*8)(*γ*), *D*_*m*_(*Fc*1, *Fc*2)(*α*), *D*_*m*_(*T*7, *T*8)(*α*), and *D*_*m*_(*C*3, *C*4)(*β*). By comparing the feature sets selected by mRMR and RFE, it seems the former is capable of removing redundancies that may exist between *D*_*m*_ and AI asymmetry features but favouring the motif ones as they provide maximum relevance. Four features overlap between the two feature selection algorithms, namely, PE(*θ*), *D*_*m*_(*Fc*1, *Fc*2)(*α*), *D*_*m*_(*T*7, *T*8)(*α*), and *D*_*m*_(*C*3, *C*4)(*β*), thus further suggesting their importance for the task at hand.

For decision fusion, in turn, the weight space was searched in steps of 0.1, and it was found that for valence, the benchmark feature set resulted in a weight of 0.2 (i.e., 0.8 for motif features), whereas a weight of 0.3 was found for arousal (i.e., 0.7 weight for motifs). Such findings highlight the importance of motif features over the benchmark ones for both valence and arousal prediction. The BACC results shown in Table 8 show the effect of score-level fusion over feature fusion. As can be seen, gains are attained only for the arousal dimension, thus further suggesting the complementarity of the two feature groups. For comparison purposes, a random voting classifier is also shown for comparison, and all attained BACCs are shown to be significantly better than chance (*p* ≤ 0.01).

Lastly, the output associative fusion method was outperformed by all other fusion methods, despite showing to be significantly better than chance. Notwithstanding, for the valence dimension, it achieved results similar to score-level fusion without the need for an exhaustive search of weights. Here, only two feature groups were explored, thus such advantage may become more critical in more complex scenarios involving additional feature groups (e.g., amplitude modulation [4]). Overall, feature-level fusion showed to be the best strategy for valence and was observed to be significantly better than score-level (*p*_val_ ≈ 0.01) and output associative fusion (*p*_val_ ≈ 0.01), whereas score-level fusion for arousal was significantly better than both feature (*p*_val_ < 0.01) and output associative fusion (*p*_val_ < 0.01). In both cases, the proposed motif features showed to provide important discriminatory information and to be complementary to existing benchmark features.

### 3.4. Comparison with Previous Work

There is increased interest in affective state recognition from EEG, and different methods have been recently proposed in the literature, many of which have also relied on the DEAP database. The work in [20], for example, explored graph-theoretic features computed from magnitude square coherence values. Such features were shown to outperform several other spectral-based and wavelet-based methods, and on the DEAP dataset, they achieved an *F*1 score of 0.63 for valence and 0.60 for arousal using an SVM classifier. For direct comparisons, the best models proposed herein achieved an *F*1 score of 0.5883 for valence and 0.6960 for arousal, thus representing a 16% increase in arousal, but a drop of 6.6% for valence. It is important to emphasize, however, that the results in [20] relied on leave-one-sample-out (LOSO) cross validation; thus, the reported results are likely higher than what are achieved with the method described herein.

More recently, in turn, the work in [4] proposed new amplitude modulation coupling features to gauge connectivity patterns as a function of valence and arousal. BACC values of 0.594 and 0.598 were reported for valence and arousal, respectively, using SVM classifiers and feature fusion, whereas somewhat lower values were attained with score-level fusion for arousal (no changes seen for valence). The values reported in [4] were obtained using a LOSO cross-validation scheme. Under the same testing setup, our proposed schemes achieve a BACC of 0.614 and 0.581 for valence and arousal, thus representing a 3.3% increase and a 2.85% decrease in performance, respectively. It is important to point out that motif-based methods did not rely on amplitude or rate of change information; therefore, fusing them with amplitude modulation features might further improve performance.

### 3.5. Study Limitations

This work has taken the first steps at gauging the advantages of motif-based features over exiting spectrum-based benchmarks. To this end, no optimization was done on the classifiers per se in order to directly compare performances achieved with the same classifier setup but with varying feature inputs. As such, it is expected that further gains may be observed not only with classifier hyperparameter optimization but also with more complex classification methods or alternate fusion schemes. The work in [20], for example, showed that relevance vector machines (RVMs) and fusion of RVMs outperformed SVMs, especially for the arousal dimension. Recent work using deep neural networks has also shown to be a promising route [79]. Future work should explore these more complex machine learning principles combined with motif-based features.

## 4. Conclusion

In this work, we propose the use of motif series and graph theoretic features for improved valence and arousal level predictions. Experiments on the widely used DEAP database show the proposed motif features outperforming several spectrum-based benchmark features. Feature-level fusion showed to provide important accuracy gains for both emotional dimensions, thus highlighting the complementarity of the two feature groups for affective state recognition. Score-level fusion, in turn, provided further improvements for arousal prediction. Overall, gains of 8.6% for valence and 9.2% for arousal could be achieved with the proposed system relative to the benchmark, and gains up to 16% could be achieved relative to prior art.

## Figures and Tables

**Table 1:** Summary and grouping of features extracted.

Feature name	No. of features	Group
(Weighted) graph features	20	Motif based features
(Unweighted) graph features	20	
Direction of flow	4	
Small-world features	12	
Permutation entropy	4	
Ordinal distance dissimilarity	48	

Spectral band power ratio	5	Benchmark spectrum based features
Shannon entropy	1	
Spectral power	4	
Asymmetry index	48	

**Table 2:** Comparison of different feature selection algorithms and number of features (nof) for benchmark feature set.

Feature groups	Valence		Arousal	
	BACC	nof	BACC	nof
ANOVA	0.5490	9	0.5316	8
mRMR	0.5404	3	0.5281	4
RFE	0.5531	3	0.5318	15

**Table 3 tab3:** Comparison of different feature selection algorithms and number of features (nof) for motif-based feature set.

Feature groups	Valence		Arousal	
	BACC	nof	BACC	nof
ANOVA	0.5818	40	0.5362	42
mRMR	0.5757	44	0.5385	20
RFE	0.5872	20	0.5500	16

**Table 4:** Comparison of different feature selection algorithms and number of features (nof) for combined benchmark-motif feature set.

Feature groups	Valence		Arousal	
	BACC	nof	BACC	nof
ANOVA	0.5930	40	0.5446	39
mRMR	0.5816	29	0.5645	17
RFE	0.6010	38	0.5598	20

**Table 5:** Top 20 features used in the best valence models for the different feature groups.

Benchmark (nof = 3, FS = RFE)	Motif (nof = 20, FS = RFE)	Combined (nof = 38, FS = RFE)
*γ*/*β*	Tr (*α*)	*C* (*α*)
*β*/*θ*	PE (*γ*)	*γ*/*β*
Spectral power (*α*)	*G* ^*w*^ (*θ*)	(*γ*+*β*)/*θ*
	*k* ^*w*^ (*θ*)	*α*/*θ*
	*C* ^*w*^ (*θ*)	*G* ^*w*^ (*θ*)
	*C* (*θ*)	PE (*γ*)
	PE (*β*)	*D*_*m*_(*P*3, *P*4) (*β*)
	*S* (*β*)	*D*_*m*_(*O*1, *O*2) (*θ*)
	*S* (*γ*)	*k* ^*w*^ (*θ*)
	*L*_*s*_ (*γ*)	Tr^*w*^ (*θ*)
	*D*_*m*_(*T*7, *T*8) (*γ*)	*C* (*θ*)
	*D*_*m*_(*Fc*5, *Fc*6) (*β*)	Spectral power (*α*)
	DoF (*θ*)	*C* (*β*)
	*D*_*m*_(*P*3, *P*4) (*β*)	*k*_*w*_ (*β*)
	*D*_*m*_(*O*1, *O*2) (*β*)	DoF (*θ*)
	*D*_*m*_(*F*3, *F*4) (*θ*)	*C* ^*w*^ (*θ*)
	Tr^*w*^ (*θ*)	AI(*C*3, *C*4) (*β*)
	*L*_*s*_ (*θ*)	AI(*P*3, *P*4) (*γ*)
	*D*_*m*_(*O*1, *O*2) (*θ*)	*D*_*m*_(*F*3, *F*4) (*θ*)
	*D*_*m*_(*F*3, *F*4) (*β*)	*D*_*m*_(*T*7, *T*8) (*γ*)

**Table 6:** Top 20 features used in the best arousal models for the different feature groups.

Benchmark (nof = 15, FS = RFE)	Motif (nof = 16, FS = RFE)	Combined (nof = 17, FS = mRMR)
(*α*+*β*)/*γ*	PE (*β*)	*D*_*m*_(*O*1, *O*2) (*θ*)
(*γ*+*β*)/*θ*	Tr (*β*)	DoF (*γ*)
AI(O1, O2) (*β*)	*C*_*s*_ (*β*)	*k* ^*w*^ (*θ*)
*γ*/*β*	*D*_*m*_(*T*7, *T*8) (*α*)	DoF (*α*)
AI(P7, P8) (*γ*)	*L* ^*w*^ (*α*)	*D*_*m*_(*T*7, *T*8) (*β*)
AI(F3, F4) (*β*)	*D*_*m*_(*FC*1, *FC*2) (*α*)	*D*_*m*_(*P*3, *P*4) (*β*)
*β*/*θ*	PE (*θ*)	*D*_*m*_(*T*7, *T*8) (*α*)
Spectral power (*β*)	*D*_*m*_(*P*7, *P*8) (*γ*)	PE (*θ*)
AI(Cp5, Cp6) (*θ*)	*L* ^*w*^ (*γ*)	*D*_*m*_(*F*3, *F*4) (*θ*)
AI(FC1, FC2) (*α*)	*C* ^*w*^ (*β*)	*D*_*m*_(*C*3, *C*4) (*γ*)
AI(P3, P4) (*θ*)	*D*_*m*_(*C*3, *C*4) (*β*)	*C* ^*w*^ (*θ*)
AI(P3, P4) (*β*)	*C* (*α*)	DoF (*θ*)
AI(C3, C4) (*θ*)	*D*_*m*_(*Cp*1, *Cp*2) (*β*)	*L*_*s*_ (*α*)
AI(Cp1, Cp2) (*α*)	*D*_*m*_(*P*3, *P*4) (*α*)	*D*_*m*_(*Fc*5, *Fc*6) (*θ*)
AI(T7, T8) (*γ*)	*D*_*m*_(*F*7, *F*8) (*α*)	*C* ^*w*^ (*α*)
	*k* (*α*)	*D*_*m*_(*Fc*5, *Fc*6) (*α*)
		*D*_*m*_(*Fc*5, *Fc*6) (*γ*)

**Table 7:** Performance comparison of different individual feature groups and subgroups.

Feature (sub) group	Valence		Arousal	
	BACC	nof	BACC	nof
Weighted graph	0.5662^*∗*^	2	0.5066	6
Unweighted graph	0.5581^*∗*^	8	0.5006	6
Small world	0.5533	6	0.5208	2
Other motif	0.5578^*∗*^	9	0.5632^*∗*^	12
Spectral power, AI	0.5400	15	0.5344	11
Power ratio	0.5467	3	0.5000	1

^*∗*^Cases where the results are significantly greater than a random voting classifier.

**Table 8:** Performance comparison of different fusion methods and a random voting classifier with chance levels.

Fusion methods	Valence BACC	Arousal BACC
Feature	0.6010	0.5645
Score	0.5875	0.5807
OAF	0.5873	0.5568
Random voting	0.5018	0.5028

## Data Availability

The DEAP database used to support the findings of this study were supplied by I. Patras under license and so cannot be made freely available. Requests for access to these data should be made to I. Patras (i.patras@qmul.ac.uk) by filling in and sending the end user license agreement at http://www.eecs.qmul.ac.uk/mmv/datasets/deap/download.html.
